# Supplementary figures and images for: Up-regulation of Grb2-associated binder 1 promotes hepatocyte growth factor-induced endothelial progenitor cell proliferation and migration
Source: PeerJ. 2019 Mar 29;7:e6675. doi: 10.7717/peerj.6675 (PMC6442669; doi:10.7717/peerj.6675)

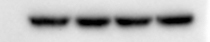

Supplement: Supplemental Information 3 — The expression of Gab1, P-Gab1, c-Met, SHP2, P-SHP2, ERK1/2, P-ERK1/2, and β-actin in each group was determined by western blot. [file peerj-07-6675-s003.zip › c-Met 2019.1.17.tif]
